# Determinants and Reasons for Dropout in Swimming —Systematic Review

**DOI:** 10.3390/sports5030050

**Published:** 2017-07-10

**Authors:** Diogo Monteiro, Luis Cid, Daniel Almeida Marinho, João Moutão, Anabela Vitorino, Teresa Bento

**Affiliations:** 1 Sport Science School of Rio Maior (ESDRM-IPSANTAREM), 2040-413 Rio Maior, Portugal; luiscid@esdrm.ipsantarem.pt (L.C.); jmoutao@esdrm.ipsantarem.pt (J.M.); anabelav@esdrm.ipsantarem.pt (A.V.); teresabento@esdrm.ipsantarem.pt (T.B.); 2 Research Center in Sport, Health and Human Development, (CIDESD), 5001-801 Vila Real, Portugal; 3 Department of Sport Science, University of Beira-Interior (UBI), 6201-001 Covilhã, Portugal; dmarinho@ubi.pt

**Keywords:** attrition, swimming, reasons, systematic review

## Abstract

The present research aims to systematically review the determinants and reasons for swimming dropout. The systematic review was conducted through electronic searches on the Web of Knowledge and PsycInfo databases from 2 February to 29 July 2015, using the keywords dropout, withdrawal, motives, reasons, sport, framework-theories, motivation, swim*, review, attrition and compliance. Fifteen studies were found and six were fully reviewed and its data extracted and analysed. Most studies were undertaken in Canada and in the United States of America (USA), and one study was conducted in Spain. Most participants were female (65.74%), and the main reasons for dropout were ‘conflicts with their trainers’, ‘other things to do’, ‘competence improvements’ failure’, ‘parents, couples or trainers’ pressure’, ‘lack of enjoyment’ and ‘get bored’. This review contributes to the present knowledge on the understanding of dropout in swimming. However, it is necessary to continue researching on this topic, validating measurement instruments and studying the motivational processes related to dropout and persistence.

## 1. Introduction

Organized sport plays an important role in children and adolescent development [1]. Despite this, there has been an increase in sports dropout in these ages, especially in occidental countries due to the massification of sport practice among youths, leading to more dropouts [2], which is particularly worrying in children aged 13–18 [3,4].

Several studies conducted in the United States of America have pointed to this tendency, indicating that about 35% of adolescents stop practicing annually [5] and that 80% of children aged 12–17 drop out of the sport programs in which they are involved, with a third of that dropping out after age 12 [6]. According to Weiss and Amorose [4], there is a decreasing number of youth aged 14–17 involved in sports when compared with those aged 5–13. This tendency also seems to appear in Australia, following evidence found in North America, since Swabey and Rogers [7] revealed that 72% of study participants drop out of sport practice in the transition from high school to college).

De Knop and colleagues [8] searched for sport tendencies in 11 European countries (Belgium, Denmark, Finland, Germany, Holland, Norway, Poland, Portugal, Spain, Sweden, United Kingdom) and found that the sport dropout rate among youths increases with age, especially in females.

Similar evidence was found in studies by Brown [9] and Guillet and colleagues [10], and this evidence is also supported by studies which equally point to high dropout rates in different sports, namely: in Ireland, a study by Trew and colleagues [11] with 20,000 young people revealed that about 20% drop out of sport completely; in France, a longitudinal study by Sarrazin and colleagues [12] with female handball players revealed that 50% of the athletes who had started practicing at the ages of 9 and 12 would drop out of the practice 3–4 years afterwards; a 10-year study by Guillet [13] in France revealed 50% dropout 2 or 3 years after the athletes started practicing; the rates increased to 75% after 5 years; also in France, a 5-year study by Trabal and Agustini [14] showed dropout rates of 45% among boxers.

It is estimated that more than one-third of all participants aged 10–17 drop out of sports practice annually, a significant percentage that represents several million youngsters across Europe and North America [4,15].

The proliferation of studies conducted in several sports [16] shows existing concern with the increasing number of athletes who drop out of sport practice [17]. However, investigations related to social and psychological factors of sports dropout are scarce [4,18] and have been shown to be crucial to understand the main reasons and/or determinants for sports dropout among youths.

The study of the sports dropout phenomenon originated descriptive studies [19] where major concern was shown over knowing what drove athletes both to practice sports and to drop out [17]. These studies led to more complex ones [12] with the goal of understanding the personal and social determinants underlying sports dropout [20]. Most studies on this topic are cross-sectional and not longitudinal (or retrospective) design.

However, a relevant point on this subject is that most authors do not define dropout specifically. Sarrazin and Guillet [21] found that its conceptual definition does not appear to be consensual in literature and, in most studies, where authors do not specify if dropout is about a specific sport or sports in general [6].

In this regard, the definition presented by Cervelló [20] seems relevant: ‘in general, sport dropout can be considered as the situation in which the person stops their sports commitment explicitly’. In this sense, with the goal of minimizing uncertainty, Gould’s [6] integrated model, in component 1 (sport withdrawal), proposes that sports dropout is viewed on a continuum ranging from a specific activity or specific program withdrawal (e.g., dropout of swimming) to a general domain withdrawal (e.g., dropout of all competitive sports permanently).

According to Sarrazin and Guillet [21], besides the main reasons that might be identified for dropout (i.e., controlled or uncontrolled reasons), individuals’ decision does not arise only as a consequence of an isolated action but from a group of reasons, more or less diversified. This is why the literature seems to be unanimous in attributing crucial importance to motivation as a predictive variable to sports dropout [4,21,22,23].

In fact, motivation appears to be responsible to explain initiation, direction, intensity and persistency of behavior [24]. Nevertheless, little is known about dropout’s motivational determinants, making it essential to recognize the reasons for dropping out, specifically to each sport, and understanding how to help athletes to persist in sports over time [25].

Over the last two decades, motivation has been one of the most studied themes in behavioral science [26], having a certain tendency for the preferential use of theoretical models (e.g., self-determination theory, achievement goal theory), which try to understand the cognitive, behavioral and emotional patterns related to sport practitioners’ goals [26].

Self-determination theory (SDT) explains the components of intrinsic and extrinsic motivation, such as the factors related to its promotion [24] considering personality factors in social contexts and the causes and consequences of self-determined behaviour [24]. According to Deci and Ryan [24], individuals’ motivation is associated with the satisfaction of the three basic psychological needs (BPN): autonomy (the need to feel independent, in that it is the individual who regulates his or her actions), competence (the need to successfully interact with the environmental stimuli), and relatedness (the need to feel connected to others or appreciated by others). These three needs are innate and universal to all human beings, which means that they are not learned and that they are relevant to human behaviour regardless of one’s gender, ethnicity, or cultural background, even considering that the means to their satisfaction can differ based on context [24]. The satisfaction of these three BPNs explains why individuals’ behaviour lies along a continuum of relative autonomy, that goes from the absence of regulation or no intention to act (amotivation), passing through more controlled forms of motivation (external and introjected regulation) until the most self-determined forms (identified and integrated regulation and intrinsic motivation).

Achievement goal theory (AGT) advocates that the cognitions, affective responses, and behaviour of people in accomplishment contexts are influenced by personal and situational factors. According to Nicholls [27], individuals are motivated to demonstrate or develop high levels of competence, basing this assessment on two orientation types (ego and task). Orientation to task is related to a conception of competence according to self-referred criteria; in other words, success means to improve and dominate the task in the individual’s sport [27]. On the other hand, orientation to ego is related to a conception of competence according to normative criteria; that is, success means to demonstrate high competence to others [25]. According to Nicholls [27], it is the motivational climate conveyed by the significant others (e.g., coach, family, friends/peers) that will determine the individual´s motivational orientation, allied with their personal characteristics.

The proliferation of investigations with these concerns, based in motivational theoretical models, has shown the impact of motivation on the person behavior in sports domain: less dropout in a specific sport [12], more persistence in a specific sport [28], better psychological state of flow of athletes [29], better group cohesion [30], and more athlete self-esteem [31].

However, studies about psychosocial factors and processes related to dropout remain scarce [18]. According to a first exploratory search made in the Web of Knowledge (WOK) with the keywords dropout and sport in the sport science and behavioral science areas, there are only 38 published studies between 1980 and 2015. This search identified only one systematic review by Crane and Temple [16], under dropout in children and youths in several kinds of sports, and observed that swimming was the second sport with more dedicated studies (following soccer).

Refining the search by adding the term swim* resulted in two studies, with no apparent systematic review which contemplates all the studies addressing dropout and/or reasons, specifically in swimming.

As stated before, and according to the exploratory search made, not much is known about dropout determinants, especially from a motivational point of view [25]; and there is a single study on the specific case of swimming [28]. On the other hand, still in this modality, most studies revealed to be descriptive and inferential [2,9,32].

Given the relevance of this issue, the present systematic study aims to elaborate a literature synthesis in order to understand the reasons and determinants for dropout in swimming, interpreting and discussing the main results and measuring instruments used, as well as the underlying theoretical framework, with the goal of providing indications for future investigations and to present eventual orientations to practice.

## 2. Methods

### 2.1. Search Strategy

The systematic review followed the PRISMA protocol for reporting systematic reviews [33], and was conducted through electronic searches on the Web of Knowledge and PsycInfo databases from 2 February to 29 July 2015, using the keywords dropout, withdrawal, motives, reasons, sport, framework-theories, motivation, swim*, review, attrition and compliance. The reference lists of the studies retrieved were examined to capture any other potentially relevant articles. The considered retreat period was between 1980 and 2015, taking into account that the first study about swimming dropout was published in 1982 [32]. Some authors were contacted directly to obtain manuscripts that were unavailable in full text in databases.

#### Inclusion/Exclusion Criteria

Inclusion criteria were: (1) publication between 1980 and 29 July 2015, (2) subjects aged 5–40 years old, (3) apparently healthy individuals, (4) written in English, (5) aimed at the reasons/motives for dropout in swimming, (6) motives reported by parents, coaches and other significant individuals about dropout in swimming.

Studies were excluded if they: (1) were published after 29 July 2015, (2) included subjects younger than age 5, (3) included populations with known diseases, (4) did not include dropout or dropout reasons in swimming, (5) were written in a language other than English, (6) reported reasons for dropout in sports other than swimming.

### 2.2. Data Extraction

A form was used to extract the following data: author, year, size, mean age or age range, design, measures, outcomes, analysis/observations, main reasons for dropout and theoretical model. The outcomes included study contributions to dropout and future research. The variables studied (size, design, measures, outcomes, analysis or observations, reasons for dropout and theoretical models) were chosen because they represent the most important variables to understand dropout.

### 2.3. Assessment of Methodological Quality of Studies

A checklist by Downs and Black [34] was used to assess the methodological quality of studies. Items that were not applied to the design of the analysed studies were removed from the 27-item checklist. The modified version consisted of items 1–4, 6, 10–13, 16–24 and 27, with the highest possible score of 19. Two main evaluators reviewed the selected studies; any discrepancies were resolved by consensus. Two assistant evaluators independently abstracted the data from each study. In the present systematic review, no study was excluded due to low quality assessment score.

## 3. Results and Discussion

### 3.1. Study Selection Process

**Figure 1:**
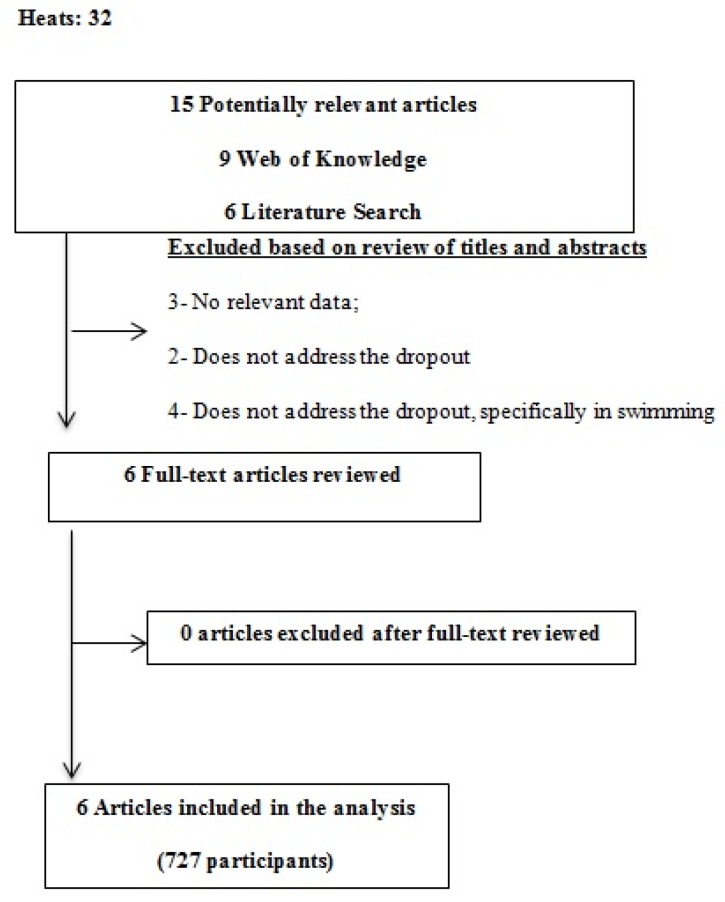
Study Flowchart 2015.

The initial search identified 32 titles in the databases, and 15 papers were retrieved as potentially relevant articles. After a review of titles and abstracts, six articles were selected, and their full text was analysed. Figure 1 presents the study selection processes.

### 3.2. Overview of the Studies

Table 1 provides a synthesis of the six studies included in this review. Four studies were undertaken in Canada [1,9,27,35], one in the United States of America [32], and one in Spain [2]. Most of the studies had only female participants (65.74%), and studies that included both genders were slightly higher in female participation (34.25%). Some studies focused solely on adolescents [1,32,35] and others on adolescents and adults [2,28]. Only Brown [9] focused on children, adolescents and adults. All studies were published between 1982 and 2008, and most were of cross-sectional design. The articles evaluated (in total) 727 subjects ages 5–30. Most of the studies met 11 or more methodological quality criteria (M = 13.57) from the 19 on the adapted checklist, suggesting good methodological quality. Only Pelletier and colleagues s [28] almost obtained the highest possible score.

The main results that came up from this systematic review revealed motivation affect behavior over time. However, the main reasons that lead to dropout in swimming seem to be associated to the lack of interest in activity and to negative aspects of environment linked to where practice occurs, including conflicts with coaches, peers and parents, and also due to lack of fun. It is important to note that most studies [2,9,32] were cross-sectional in design and used descriptive and inferential analysis. Furthermore, they all had a common dropout reason (‘had other things to do’), although in the Brown [9] study this reason was subentended on the following ones: ‘swimming was no longer important compared with other activities’/’desire to participate in other activities’. This reason came up regardless of age and gender as evidenced in several studies [17].

Curiously, the Salguero and colleagues [2] study made in Spain shares a reason with the Gould and colleagues [32] study—‘did not have enough fun’—and both studies show females value the reason ‘did not like the pressure’ more than males. Despite the similarities found in these two studies, however, they reached distinct conclusions. Gould and colleagues [32] presented reasons such as ‘I wanted to play another sport’ and ‘I did not like the pressure’, while Salguero and colleagues [2] included reasons such as ‘my skills did not improve’, ‘the training was too hard’ and ‘it was boring’. This shows that although the reasons are different, they are controllable by the individual.

Nevertheless, the Gould and colleagues [32] study revealed age differences. The age 15–18 group valued reasons such as ‘no team work’, ‘parents or friends did not want me to’ and ‘not enough challenge’ more than the age 10–14 group. The Salguero and colleagues [2] study did not report age-related differences.

Both studies analysed differences related to years of experience, leading to distinct conclusions. Gould and colleagues [32] reported that the main dropout reason of less-experienced athletes was ‘I was not with my friends’ while Salguero and colleagues [2] reported that the main dropout reason for these athletes was ‘interest in another sport’. It is important to note that these two studies used the same questionnaire, “Questionnaire of Reasons for Attrition in Swimming” [32], and neither used a theoretical support model.

In another perspective, the Brown [9] study pointed out divergent reasons from the other transversal studies: ‘interest conflicts with parents, friends and coach’, ‘desire to spend more time with friends’, ‘lack of success’ and ‘need to choose between available alternatives’. This may be due to the fact that this study only had female participants, as it has been demonstrated in some studies [10]. The Brown study [9] did not report differences in age-related or years of experience reasons.

However, the Brown [9] study came up with one dropout reason (‘interest conflicts with parents, friends and coach’), similar reasons were found by Fraser-Thomas and colleagues [35] in another qualitative study: ‘less one-on-one coaching’, ‘throughout development’, ‘pressuring parents during adolescence’ and ‘lack of swimming peers during adolescence’.

‘Less one-on-one coaching’ appears in the two qualitative studies about swimming dropout made by Fraser-Thomas and colleagues [1,35] but different reasons also emerged. In Fraser-Thomas and colleagues [1], the main dropout reasons were ‘involved in fewer extra-curricular activities’, ‘less unstructured swimming play’, ‘had parents who were high-level athletes in their youth’, ‘were more likely to be youngest in their training group’ and ‘were less likely to have a best friend at swimming’. In Fraser-Thomas and colleagues [35], the main dropout reasons were ‘spoke of early performance’, ‘pressuring parents during adolescence’, ‘lack of swimming peers during adolescence’ and ‘siblings rivalries’.

The analysis also highlighted that these reasons are different from those cited in the transversal studies except for one identified by Brown [9]: (‘interest conflicts with parents, friends and coach’). This probably is due to the fact that these studies adopted a qualitative methodology, favouring open questions and dealing with data by categories (content analysis) afterward.

Lastly, the only longitudinal study contemplated in this review [28] did not report concrete dropout reasons, but what determines dropout. In this study, it seems that motivation is the key variable that works as a dropout predictor, as suggested by several studies [21,23]. In fact, the Pelletier and colleagues [28] study seems to indicate that amotivation (i.e., absence of behaviour regulation) had a positive predictive effect on dropout.

It is important to underline that, in this study, the authors resorted to a more robust analysis method (structural equation modeling), enabling them to predict the variables’ impact on each other. This established hipotetic causal relationships based on self-determination theory (SDT) [24] made it possible to simultaneously assess dropout determinants and persistence using a longitudinal methodology through which a follow-up was made to understand if they dropped out or persisted in swim practice over time.

Only four of the studies in this review used underlying theoretical support models [1,9,28,35]. Brown [9], using a conceptual model based on sociological theories, perceived the factors that influence competition dropout. According to the author, it appears that sports (de)socialization, swimming in this case, occurs over time and at the same time that the individual stops investing/identifying himself with his sport’s role.

Although, in this way, there seems to be a group of factors responsible for dropout in youth sports, Brown [9] concluded that there were not enough data to establish a connection of cause–effect. This is in line with Cervelló and colleagues [25] who said little is known about dropout determinants.

The Fraser-Thomas and colleagues [1,35] studies used a model already known in the sports area: the developmental model of sport participation (DMSP) developed by Côte and colleagues [36]. This model divides sports participation into three phases: sampling years (6–12); specializing years (13–15) and investment years (16+). The authors examined the part played by significant others (e.g., coaches, parents, friends and siblings), trying to understand their influence on dropout and engagement of swimmers according to DMSP′s phases. This approach evidences the importance of environmental factors on dropout or persistence of athletes, specifically, the motivational climate induced by significant others.

Fraser-Thomas and colleagues [1,35] studies are according to the theoretical assumptions recommended by achievement goal theory (AGT) [27], empirically comproved by several sports dropout studies based in this theoretical model [12,25].

Specifically, Fraser-Thomas and colleagues [1] aimed to understand how physical factors (e.g., training patterns, level of maturation) and psychosocial factors (e.g., parents, coaches, peers, siblings) might contribute to dropout and prolonged engagement. They concluded that there are differences at both the physical factor level (e.g., less extra-curricular activities, less unstructured swimming play) and the psychosocial factor level (e.g., parents’ support, less one-on-one coaching) for athletes who dropped out compared with those who persisted.

Furthermore, this study went one step beyond past studies by collectively examining training, maturational and psychosocial factors throughout development that might contribute to dropout or prolonged engagement rather than focusing on specific variables at one stage of development. However, Fraser-Thomas and colleagues [1] said further studies need to be developed with other factors (physical, psychosocial and motivational) that explain how they contribute to dropout or persistence.

At last, the longitudinal study of Pelletier and colleagues [28], which used SDT as a theoretical framework, suggested that motivation affects behaviour over time, whether in the short, medium or long term. Notwithstanding, authors concluded that lack of motivation seems to be a dropout predictor.

Remaining studies [2,32] did not use a theoretical support model, despite presenting different reasons for dropout. However, they seem to reach a common contribution: in order to minimize dropout risks created by significant others, it is necessary to modify athlete’s environmental conditions. In other words, the significant others (e.g., coaches) play a crucial part in creating propitious conditions for athletes to satisfy their needs in practise and persistence in sports [2,32].

Such recommendations seem to be in accord with what many authors stand for: that most reasons for dropout are controllable by the athlete [6,21] and that motivation works as a dropout predictive variable [21,23].

## 4. Conclusions

Taking into account the main purpose of the present systematic review, the results clarify that regardless of the design type of each study, it appears to be unanimous that the main reasons leading to dropout in swimming are controllable by the athletes (e.g., ‘conflicts with coaches’, ‘having other things to do’, ‘failure in competence improvement’, ‘pressure by the parents, peers or coaches’, ‘lack of fun’, ‘boredom’). Thus, attention should be paid to this aspect because the key to prevent dropout probably is related with its determinants.

As observed, there still is a lack of knowledge about dropout determinants, which makes it necessary to develop studies based on theoretical motivational models, especially AGT and SDT. This type of study is needed to evaluate the motivational climate (e.g., task and ego-involving) supported by significant others (e.g., parents, peers and coaches) but also because the factors related with development are addressed in the studies included in this review. These types of studies are also needed because there appears to be strong empirical evidence that behavioral regulation (i.e., autonomous motivation vs. controlled motivation) works as a variable to predict dropout, as demonstrated by several authors [28].

From this analysis, it appears that another of the knowledge gaps is related to quantitative measurement models (i.e., questionnaires) which assess the reasons for dropout. The most common instrument used for this purpose is the questionnaire of reasons for attrition (QRA), which was translated into Spanish and validated in an exploratory factor analysis by Salguero and colleagues [37] and recently into Finnish by Rottenstein and colleagues [38]. However, measurement model of QRA never was validated through a confirmatory factor analysis. Nevertheless, QRA is the only questionnaire which assesses reasons for dropout after athletes completely stop their participation in sport and was developed specifically for swimming.

At last, it is important to develop studies which assess the determinants of persistence across time span of swimming practice so that, as Cervelló and colleagues [25] suggested, through these variables strong strategies for dropout prevention may be developed.

The results of this review could have important implications because this study complements others studies [16], making a contribution to identify dropout reasons and their determinants in a specific sport and its dissemination, alerting for the importance of dropout prevention as suggested by Gould [6]. This study also makes it clear that the main reasons for dropout are controllable by the subject (e.g., ‘had other things to do’). Therefore, future studies can be developed in order to understand the impact of motivational climate (task and ego-involving), basic psychological needs, motivation regulation and consequences (e.g., persistence, dropout and burnout), based on the hierarchical model of intrinsic and extrinsic motivation proposed by Vallerand [39,40].

The results of this study could have some practical orientations, especially for coaches or teachers: (i) practice or training process must be fun and not just focus on development of physical and technical skills (i.e., training diversity); (ii) planning keeping in mind other activities in which the swimmer is involved (e.g., have periodical reunions with legal guardians), it is important to privilege relationships among parents, coaches and athletes; and to project swimming as a promotor of academic return (i.e., great swimmers and good students); (iii) training process must be more task-oriented and less result-oriented, coaches/teachers must create conditions to an autonomous supportive climate that promotes athletes’ satisfaction of autonomy and competence, and also leads to identification with sport and to feelings of pleasure; (iv) swimmers must feel their coaches/teachers are a reference, and coaches/teachers need to pay attention to them, with no exception, independently of their physical or technical quality.

To conclude, this review was another step toward dissemination of dropout phenomenon which needs to be understood in time and space, making it an important constant study in several contexts because there is an increase in dropout number of organized sports. It is necessary to continue the research in this area, validating measurement instruments and studying motivational processes underlying both dropout and persistence.

## Figures and Tables

**Table 1:** Characteristics of the studies included in the review 1980–2015.

Author, Year	Country	N	Mean Age or Age Range (Years)	Design	Measures	Outcomes	Analysis/Observations	Dropout Reasons	TM	QS
**Gould, Feltz, Horn and Weiss (1982)**	USA	50 Swimming (29 M; 21 F)	10–18	Mixed methodology with questionnaire and interview	QRA and interview with 5 areas *	The outcomes revealed that the majority of youth swimmers who discontinue participation do so because of interest in other activities, and not because of excessive pressure, a lack of fun, and/or over emphasis on winning. It was noted, however, that these more negative reasons cause some swimmers in some situations.	Descriptive and univariate analyses	Had other things to do; I was not as good as I wanted to be; I did not have enough fun; I wanted to play another sport; I did not like the pressure.	-	13
**Brown (1985)**	Canada	211 former swimmers and 193 currently swimmers (F)	Different age groups, between starting age (5–7 years old) until 30 years old	Cross-Sectional	“A copy of the questionnaire used is available from the author upon request”	The findings of the present study provide preliminary evidence that resocialisation from a sport role is a process occurring over time as individuals gradually divest themselves of investing and identifying with the sport’s role. Furthermore, and in contrast to early notions of withdrawal from youth sport roles, a variety of factors related to differential socialization seem important in the process. However, the data in the present study does not provide an adequate basis on which to assess cause and effect.	Bivariate analysis	Interest conflicts with parents, friends and coach; the desire to spend more time with friends; the fact that swimming was no longer important compared with other activities; the desire to participate in other activities; lack of success; need to choose between available alternatives.	Conceptual Framework	13
**Pelletier, Fortier, Vallerand and Brière (2001)**	Canada	369 competitive swimmers (174 M; 195 F)	13–22	Prospective Study	PIB; SMS and persistence **	The outcomes of this study revealed that all types of motivation affect the behavior along time (short, medium and long).	SEM	This study revealed that amotivation predicted dropout positively.	SDT	18
**Salguero, Gonzalez-Boto, Tuero, and Márquez (2003)**	Spain	62 (40 M; 62 F)	14–30	Cross-Sectional	QRA	Negative factors related with the aspects of the athletic environment that lead to discontinuing in competitive swimming.	Descriptive and univariate analyses	Had other things to do; my skills did not improve; the training was too hard; did not like the coach; not enough fun and it was boring	-	12
**Fraser-Thomas, Côté and Deakin (2008a)**	Canada	25 dropout (21 F; 4 M) and 25 engaged swimmers (21 F; 4 M)	13–18	Qualitative study with a retrospective interview	All measures were collected through a swimming adapted version of the Côté, Ericsson and Law´s (2005)	This study went one step beyond past studies by collectively examining training, maturational, and psychosocial factors throughout development that may contribute to dropout and prolonged engagement, rather than focusing on specific variables in one stage of development.	Correlations and Multivariate analysis.	Involved in fewer extra-curricular activities, less unstructured swimming play, and received less one-on-one coaching throughout development; several developmental milestones (started training camps, started dry land training and were top in club), and more likely to have had parents who were high-level athletes in their youth, were more likely to be youngest in their training group and were less likely to have a best friend at swimming.	DMSP	14
**Fraser-Thomas, Côte and Deakin (2008b)**	Canada	10 dropout (8 F; 2 M) 10 engaged swimmers (7 F; 3 M)	13–18	Qualitative interview	Qualitative interview aimed to gain in-depth understanding of participants’ swimming involvement so as to paint a full picture of athletes’ development. ***	Identification of the reasons for dropout of the training standards and the role of significant others (e.g. coaches, parents, peers and siblings) with a qualitative research and contribution for the dropout explanation in these areas.	All interviews were digitally transcribed from verbatim.	Spoke of early performance; limited one-on-one coaching; pressuring parents during adolescence; lack of swimming peers during adolescence and rivalries among siblings.	DMSP	13

Legend: M = male gender; F = female gender; TM = theoretical models; QS = quality score; *= other activities, encouragement offered by parents and coach, what they liked and disliked about competitive swimming and coach and reasons for initially joining and discontinuing participation; QRA = questionnaire of reasons for attrition; - = information’s not reported by authors; PIB = perceived interpersonal behaviors; SMS = sport motivation scale; SEM = structural equation modelling; DMSP = development model of sport participation; SDT = self-determination theory; **to assess the persistence all participating swimming teams were contacted in order establish a list of persistence and dropout; *** = for each development stage of swimming involvement, athletes were questioned in five areas: training patterns, parent, coach and sibling influences.
